# Dual and Single-species Nematode Infections Distinctly Modulate Defense Metabolism in *Brassica nigra* Roots

**DOI:** 10.1007/s10886-025-01637-8

**Published:** 2025-09-10

**Authors:** Jessil Ann Pajar, April Lyn Leonar, Pius Otto, Franziska Sabine Hanschen, Stefanie Döll, Nicole M. van Dam

**Affiliations:** 1 Leibniz Institute for Vegetable and Ornamental Crops (IGZ) e.V., Großbeeren, Germany; 2 Max Planck Institute for Chemical Ecology, Jena, Germany; 3 German Centre for Integrative Biodiversity Research (iDiv), Jena-Halle-Leipzig, Germany; 4 Institute of Biodiversity, Ecology and Evolution (IBEE), Friedrich-Schiller-University, Jena, Germany; 5 Institute of Food Technology and Food Chemistry, Technische Universität Berlin, Berlin, Germany

**Keywords:** Glucosinolates, Nematode-nematode interactions, Plant defense, Root metabolome, Simultaneous herbivory, Systemic induced responses

## Abstract

Plant roots are exposed to various organisms that significantly impact plant productivity. Plant-parasitic nematodes (PPNs) such as *Meloidogyne* spp. and *Pratylenchus* spp. are microscopic roundworms that damage several crops. In natural populations, *M. incognita* and *P. penetrans* were found to infest black mustard (*Brassica nigra*) plants simultaneously. Considering their different feeding strategies and contrasting effects on plant defense responses, we hypothesized that dual infection may affect each nematode’s performance via changes in the root metabolome. Using untargeted and targeted metabolomics, we evaluated how single and dual nematode infections affected *B. nigra* root metabolome. We combined these metabolic data with measures of early infection success. At three days post-inoculation, dual infection increased *M. incognita* penetration success, while that of *P. penetrans* remained unaffected. Compared to single-species infections, dual infections resulted in distinct root metabolic changes by reducing indole glucosinolates (GSL), gluconasturtiin, lignans, and phenylpropanoids. Dual and single-species infections affected different GSL classes. The allyl GSL, sinigrin and its breakdown products increased in *P. penetrans*-infected plants, while gluconasturtiin and 2-phenylethyl ITC increased in *M. incognita*-infected plants. This shows that plant defense response to dual nematode infection differ from those of single species, which has consequences to the early infection success of each nematode species.

**Supplementary Information:**

The online version contains supplementary material available at 10.1007/s10886-025-01637-8.

## Introduction

Plant-parasitic nematodes (PPNs) are root-feeding roundworms that pose significant threats to global agriculture. Several PPN species are causing extensive damage to crop plants (Nicol et al. 2011; Jones et al. 2013; Tileubayeva et al. 2021), with yield losses estimated at 173 billion USD worldwide (Kantor et al. 2022). *Meloidogyne incognita* and *Pratylenchus penetrans* are two prominent PPN species known for their detrimental effects on various crops (Jones et al. 2013; Kantor et al. 2022; Zhang et al. 2023). The root-knot nematode (RKN) *M. incognita* is a sedentary endoparasitic nematode. The infective juvenile (J2) invades the roots by moving in-between cells until it reaches the vascular cylinder and induces the formation of giant cells. Thereafter, it becomes sedentary throughout its feeding and reproductive stages (Kyndt et al. 2012a; Bartlem et al. 2014; Rutter et al. 2022). The giant cells serve as nutrient sinks, diverting resources away from other plant tissues and impairing its growth and productivity (Kyndt et al. 2013; Rutter et al. 2022; Mitchum et al. 2023). Additionally, *M. incognita* secretes effectors to modulate its host’s immune responses and facilitate parasitism (Gheysen and Mitchum 2011; Eloh et al. 2016; Rutter et al. 2022). *Pratylenchus penetrans*, on the other hand, is a migratory endoparasitic nematode that feeds by puncturing individual cells and withdrawing nutrients as they move through the roots (Sijmons et al. 1994; Fosu-Nyarko and Jones 2016). The effectors secreted by *P. penetrans* enable efficient nematode mobility through root cells, causing tissue destruction due to, for example, cell wall degrading enzymes (Vieira et al. 2018). Unlike RKNs, *P. penetrans* does not induce the formation of giant cells, but it can cause extensive root damage leading to root necrosis, reduced nutrient and water uptake, as well as render the infected roots vulnerable to secondary microbial infections (Sijmons et al. 1994; Fosu-Nyarko and Jones 2016). Several studies showed that plants can recognize and respond to nematode infections through various defense mechanisms. This includes activating defense-related genes and synthesizing secondary metabolites (Hofmann et al. 2010; Kyndt et al. 2012a; Desmedt et al. 2020). Studies on related PPNs demonstrated that migratory and sedentary endoparasitic nematodes regulate plant defenses differently (Lohmann et al. 2009; Kyndt et al. 2012b). In rice, early root infection by the migratory endoparasitic nematode *Hirsmaniella oryzae* up-regulated biotic stress-responsive genes, inducing oxidative stress and programmed cell death. In contrast, early infection by the sedentary endoparasite, *Meloidogyne graminicola*, suppressed root defense-responsive genes (Kyndt et al. 2012a).

*Brassica* spp. plants are frequently challenged by PPNs (Liébanas and Castillo 2004; Mukhopadhyay and Roy 2006; Hol et al. 2016). In a survey by Hol et al. (2016), *Meloidogyne* spp. and *Pratylenchus* spp. were found to infest field-grown black mustard plants, *Brassica nigra*. *Brassica* plants are known to deploy glucosinolates as defense response against nematodes (van Dam et al. 2018; Desmedt et al. 2020). Glucosinolates (GSLs) are the main defense compounds of plants in the order Brassicales (Tsunoda et al. 2017; Touw et al. 2020). They are β-D-thioglucosides that can be classified by differences in their side chains and are grouped into aliphatic, benzenic, or indolic GSLs (Kliebenstein et al. 2001; Agerbirk and Olsen 2012). Analyzing GSL profiles of nine GSL-producing plants including *B. nigra*, Tsunoda et al. (2017) showed that root GSL concentrations are significantly higher than in the shoots. Other than shoots, roots contain high levels of gluconasturtiin (2-phenylethyl GSL) (Tsunoda et al. 2017; Touw et al. 2020; Sontowski et al. 2022), which serves as defense against nematodes (Potter et al. 1998) and insect herbivores, such as *Delia radicum* and *D. floralis* (Sontowski et al. 2022). Several GSLs, either applied as plant extracts or incorporated as biofumigants, can reduce PPN populations and associated symptoms (Ren et al. 2018; Yu et al. 2019; Dahlin and Hallmann 2020; Eugui et al. 2022).

Beyond the localized defense response, GSLs and other plant secondary metabolites are induced systemically in response to nematode infections, which correlated with altered performance of other herbivores feeding on the same plant (Hol et al. 2016; van Dam et al. 2018; Pajar et al. 2024; Touw et al., 2025). Plant-mediated interactions between nematodes and aboveground insects, such as aphids and caterpillars (Hol et al. 2016; van Dam et al. 2018; Mbaluto et al. 2020), and on root-feeding insects such as *D. radicum*, have been reported (Touw et al., 2025). However, most studies have focused on responses to infections by single PPN species. This overlooks the fact that in natural environments, multiple PPN species interact within the same host plant (Hol et al. 2016; Mateille et al. 2020). Sedentary and migratory PPN species may antagonize each other, whereby the population increase of one species suppresses the other (Gay and Bird 1973; Chapman and Turner 1975; Fontana et al. 2015). It has been suggested that such effects are caused by competition for feeding sites or resources (Mateille et al. 2020). Alternatively, antagonistic relationships between PPNs may be governed by plant-mediated mechanisms. For example, the phytohormones salicylic acid (SA) and jasmonic acid (JA) can be activated during PPN infections, which mainly depends on the PPN species interacting with the plant (Kyndt et al. 2012a; van Dam et al. 2018; Gheysen and Mitchum 2019). In general, SA is associated with systemic acquired resistance and is effective against biotrophic pathogens and sedentary PPNs (Bonnet et al. 2017; Gheysen and Mitchum 2019). On the other hand, JA is involved in induced responses that are typically more effective against necrotrophic pathogens and migratory PPNs (Bonnet et al. 2017). Commonly, JA and SA act antagonistically via phytohormonal crosstalk (Pieterse et al. 2009; Bonnet et al. 2017; Gheysen and Mitchum 2019). When a plant is simultaneously infected by several PPNs, JA-SA crosstalk may lead to a modified metabolic response, as it was found for chewing and phloem-feeding insects aboveground (Bonnet et al. 2017). This also prompts the question of whether the plant’s defense response against concurrent (dual) nematode infections differs from those activated during single-species infections.

In this study, we examined the changes in the root metabolome of *B. nigra* when the plant is challenged by PPNs with contrasting feeding strategies: the migratory endoparasitic nematode *P. penetrans*, and the sedentary endoparasitic nematode, *M. incognita.* We hypothesized that the root metabolic changes in response to each nematode species are distinct due to their different feeding strategies. Moreover, we also analyzed root metabolic changes in response to the concurrent infection of *M. incognita* and *P. penetrans.* We hypothesized that simultaneous infection would attenuate the defense response in comparison to single-species infections. Thus, the performance of one or both nematodes would improve on plants with simultaneous or prior infection by the other nematode. To test these hypotheses, we performed untargeted metabolic analysis on root samples infected with each nematode species as well as on root samples with concurrent infection. Our results indicate that root metabolic changes in response to concurrent infection by *M. incognita* and *P. penetrans* are distinct from those observed in single-species infections, in particular for GSLs, lignans, and phenylpropanoids. Targeted analyses of GSLs and their breakdown products showed that sinigrin and allyl isothiocyanate (ITC) levels increased in *P. penetrans*-treated plants, while gluconasturtiin and 2-phenylethyl ITC marginally increased in *M. incognita*-infected plants. These GSLs and ITCs were reduced in MP-treated plants, along with indole GSLs, lignans and phenylpropanoids. Concurrent infection increased the number of *M. incognita* inside the roots, while *P. penetrans* numbers remained unaffected. However, fewer *P. penetrans* were found in the roots when inoculated two days after *M. incognita*. Our results highlight the importance of studying plant response to multiple nematode infections, as this may lead to changes in plant defense mechanisms that differ from those observed in single-species infections.

## Materials and Methods

### Plant Growing Conditions

*Brassica nigra* seeds were bulk-collected from a wild population at Elderveld, Arnhem, the Netherlands, in 2005. Prior to germination, the seeds were washed with 1% sodium hypochlorite solution and rinsed with ultrapure water. These clean seeds were germinated in water-soaked glass beads in plastic containers. The containers were covered with transparent plastic lids and kept in a climate chamber in a 16:8 (light: dark) photoperiod at 20:16° C (day: night). The seeds were germinated for 10 days before transplanting in sand pots (for the root material sampling) or Pluronic gel plates (for nematode-nematode interaction assay). The plant pots were prepared and maintained following the protocols described by van Dam et al. (2003). Before transplanting, each pot was filled with 2.5 l of dry, heat-treated sand (90 °C for 1 h) and supplied with 200 ml tap water. The plants were grown in a greenhouse at 16:8-hour photoperiod, minimum light intensity 300 µmol m^−2^ s^−1^; average temperature 25 °C; 60–80% relative humidity. The plants were supplied with 100 ml half-strength 3P Hoagland solution weekly. The developmental stages of *B. nigra* were monitored following the universal BBCH scale (Lancashire et al. 1991).

### Nematode Cultures

*Pratylenchus penetrans* was provided by the Plant Science Research Unit, Research Institute for Agriculture, Fisheries and Food (ILVO), Merelbeke, Belgium. The culture was maintained in carrot discs at 25 ± 1 °C. *Meloidogyne incognita* was provided by Bejo, Warmenhuizen, the Netherlands and was maintained on *Solanum lycopersicum* cv. ‘Moneymaker’ under greenhouse conditions (16:8 photoperiod at 25 ± 3° C). All infective stages of *P. penetrans* (juveniles and adults) and J2s of *M. incognita* were extracted from the cultures via modified Baermann technique (Hooper et al. 2005). A solution containing a specified number of nematodes in water-Tween20^®^ solution (0.04% v/v) was prepared and used for inoculation.

### Greenhouse Experiment

#### Nematode Inoculation

To investigate nematode-induced changes in the roots, plants were assigned to the following treatment groups: control, *M. incognita* only (Mi), *P. penetrans* only (Pp), *M. incognita + P. penetrans* inoculated simultaneously (MP). Four-week-old (BBCH 32) *B. nigra* plants were inoculated with 2 ml of nematode suspension, each containing 200 nematodes in the infective stage in water-Tween20^®^ solution. The same number of nematodes was applied in MP-treatments with 100 J2s of *M. incognita* and 100 infective stages of *P. penetrans* in 2 ml solution. Control plants were mock-inoculated with water-Tween20^®^ solution. The nematode suspension was introduced into a small hole near the roots. Thereafter, 50 ml of tap water was added to facilitate nematode dispersal. The nematodes were allowed to infect for 10 days. A separate group of nematode-infected plants were left to grow for sixteen days more (total = 26 days) to see the root galls and/or lesions, thereby confirming the success of nematode infection (Fig. S1).

### Root Sampling

On the tenth day-post nematode inoculation (10 dpi), whole root samples were collected for metabolic (*n* = 3–5) and gene expression (*n* = 3–4) analyses. Plants were carefully lifted from their pots, and the roots were washed under running water. Excess water was drained, and the samples were collected, flash-frozen in liquid nitrogen, finely pulverized and stored at −80 °C freezer until further use.

### Untargeted Metabolomics Via LC-MS Coupled with MS/MS

Root metabolites were extracted using Liquid Chromatography-Time of Flight-Mass Spectrometry (LC-ToF-MS) according to Weinhold et al. (2022). Twenty milligrams of ground freeze-dried root sample was mixed with 1 ml extraction solution (25% acetate buffer, pH 4.8 and 75% HPLC-grade MeOH). The mixture was placed in an ultrasonic bath (5 min, 30 Hz) then centrifuged at 15,000 x g for 15 min at room temperature. The supernatant was transferred to a new 2 ml Eppendorf Tube^®^ while the pellet was re-extracted with 1 ml of extraction solution, placed in ultrasonic bath (5 min, 30 Hz) and centrifuged again at 15,000 x g for 15 min at room temperature. The supernatant was combined with that from the first extraction, then centrifuged at 15,000 x g for 10 min. Afterwards, 200 µl of supernatant was transferred in an HPLC vial and was added with 800 µl of extraction solution.

The LC-MS analysis was conducted on an UltiMate™ 3000 Standard Ultra-High-Performance Liquid Chromatography system (UHPLC, Thermo Scientific) with an Acclaim^®^ Rapid Separation Liquid Chromatography (RSLC) 120 column (150 mm × 2.1 mm, particle size 2.2 μm, ThermoFischer Scientific). The detailed instrument settings and post-processing parameters are provided in Method S1. After processing and blank feature-subtraction, the dataset contained 5,919 features. The resulting features with MS/MS data were annotated using an in-house spectral library. All detected features were classified and formatted using SIRIUS/CANOPUS (Dührkop et al. 2019, 2021; Kim et al. 2021) and MetIgel v.1.0 ©Smith and Schedl, 2021.

### Targeted Glucosinolate Analysis

The glucosinolates (GSLs) were quantified via High-Performance Liquid Chromatography (HPLC, UltiMate™ 3000, Thermo Scientific) using 50 mg freeze-dried ground root sample following the protocol by Grosser and van Dam (2017). The acquired data was further processed in Chromeleon 7.2 SR5 MUa (9624; Thermo Fisher Scientific, Waltham, MA, USA). Identification of GSLs was based on comparison of retention time and UV spectra from commercially available reference standards, as described in detail by Touw et al. (2020).

### Quantification of GSL Breakdown Products

Glucosinolate breakdown products were extracted following Hanschen and Schreiner (2017). Briefly, 25 mg of freeze-dried ground root sample was weighed into extraction vials and left to hydrolyze with 250 µl ultrapure water for 1 h. Dichloromethane (DCM, 2 ml) was added along with 100 µl DCM containing 0.2 µmol benzonitrile (internal standard). The solution was shaken and centrifuged. The resulting DCM extract was dried over Na_2_SO_4_. The extraction was repeated twice, adding only 1.5 ml DCM to the last two sets. The extracts were combined and reduced under nitrogen steam to 300 µl. The GSL breakdown products were quantified via Gas Chromatography-Mass Spectrometry (GC-MS, Agilent 7890 A Series GC System, Agilent Technologies) equipped with an Agilent 7683 Series Autosampler, an Agilent 7683B Series Injector and an Agilent 5975 C inert XL MSD, using the settings as in (Hanschen 2024).

### Gene Expression Analysis

Representative GSL biosynthesis and transport genes, and genes involved in phytohormone-mediated defenses were analyzed to assess the involvement of the respective processes in plant-nematode interactions. The genes and their short descriptions are as follows: *CYP83A1* (CYTOCHROME P450, FAMILY 83, SUBFAMILY A, POLYPEPTIDE 1) is involved in the biosynthesis of aliphatic and benzenic GSLs. *CYP79B2* (CYTOCHROME P450, FAMILY 79, SUBFAMILY B, POLYPEPTIDE 2) is involved in the conversion of tryptophan to indole-3-acetaldoxime, a precursor to IAA and indole GSLs; *MYB122* (transcription factor MYB122) is known to regulate indole GSL biosynthesis; *PR1* (pathogenesis-related protein 1) is a salicylic acid-responsive gene; *PAL1* (phenylalanine ammonia-lyase 1) involved in the first reaction in the biosynthesis of secondary metabolites from L-phenylalanine; *ERF1* (ethylene response factor 1) encodes a transcription factor that can be activated by ethylene or jasmonates. The list of primer sequences used in this experiment is given in Table S1. Total RNA was extracted from 100 ± 5 mg ground frozen root tissue following a protocol adapted from Oñate-Sánchez and Vicente-Carbajosa (2008) as described in detail by Touw et al. (2020). The quality of DNAse I (Thermo Scientific, Waltham, MA, USA)-treated RNA was visually evaluated by gel-electrophoresis and by measurement of 260/230 nm and 260/280 nm absorbance ratios using a NanoPhotometer^®^ P330 (Implen, Munich, Germany). Stable cDNA was synthesized from 4 µg purified total RNA using Revert Aid H minus reverse transcriptase (Thermo Scientific, Waltham, MA, USA) following the manufacturer’s instructions. The samples were incubated at 42 °C for 60 min, 50 °C for 15 min, and finally, 70 °C for 15 min in a thermal cycler (Techne, Stone, UK). Real-time quantitative polymerase chain reaction (RT-qPCR) was performed using the CFX384 Real-time system (BioRad, Munich, Germany) with gene-specific primers (Table S1). The qPCR conditions were: 2 min at 50 °C, 10 min at 95 °C, and 40 cycles of 15 s at 95 °C and 1 min at 60 °C. Three technical replicates were analyzed per gene for each biological replicate (*n* = 3–4). The data was normalized to the average expression of the housekeeping genes *GAPDH* and *TIP41*. The relative expression of target genes was calculated using the 2^−ΔΔCT^ method as described in Livak and Schmittgen (2001).

### Nematode-nematode Interaction Assay

Root attraction assays were performed using a Pluronic gel medium, which simulates the three-dimensional soil environment (Wang et al. 2010). Following the protocol by Wang et al. (2009), the nematodes were mixed evenly into the Pluronic gel (Pluronic F-127, Sigma-Aldrich). One ml of gel was poured onto 3-cm Petri plates with 100 nematodes per plate. The plates were incubated at 27 °C for the gel to slightly solidify. The treatments were as follows: control (+ water), *M. incognita* (Mi), *P. penetrans* (Pp), and Mi + Pp inoculated simultaneously (MP) (*n* = 10). To address the ambiguity of which PPN species infects the plant first, we included a sequential infection to see if prior infection of one species affects the other. For this, we added the treatments *M. incognita* with *P. penetrans* pre-infected plant (Mi after Pp, *n* = 10) and *P. penetrans* with *M. incognita* pre-infected plant (Pp after Mi, *n* = 10).

One ten-day old *B. nigra* seedling was placed in the gel plate. In plates with pre-infection (Mi after Pp; Pp after Mi), the first nematode species was allowed to infect for 48 h, whereafter the seedling was transferred to a plate containing the nematode of interest. Nematodes touching the roots were counted after 4, 24, 48 and 72 h. After counting at 72 h, the roots were stained with acid-fuchsin (Bybd et al. 1983) to count the nematodes that have entered the roots. For single-species plates (Mi alone, Mi after Pp, Pp alone, and Pp after Mi), we divided the number of nematodes by 100. For the data from mixed-species plates (MP), we focused on the number of each species: Mi on MP refers to *M. incognita* counts in mixed plates, while Pp in MP refers to *P. penetrans* counts in mixed plates. The number of nematode of interest from these mixed plates were divided by 50.

*M. incognita* and *P. penetrans* have different feeding strategies and infection timelines. To facilitate uniformity in the context of concurrent infection, we defined early infection as the period from invasion until feeding site establishment for *M. incognita*, and for *Pratylenchus* spp. the period before complete invasion of the vascular tissues, including early penetration and migration, which can occur three hours to three weeks post-inoculation (Acedo and Rohde 1971; Hol et al. 2016; van Dam et al. 2018).

### Statistical Analyses

After data processing and compound annotation of the LC-MS data, the feature tables were exported to MetaboAnalyst 6.0 (Pang et al. 2024). The dataset was filtered based on their Interquartile Range. Given unequal replicate numbers and inherent variability among plants, Pareto-scaling was employed in addition to log_10_-transformation prior to the data analysis. The resulting dataset was analyzed using principal component analysis (PCA), followed by *Permutation Multivariate Analysis of Variance* (PERMANOVA). Differentially abundant (DA) features based on pairwise comparisons between the control and each of the nematode-treated plants (Mi, Pp, MP) were identified using the Volcano Plot function in MetaboAnalyst 6.0. The lists were exported to InteractiVenn, creating Venn diagrams (Heberle et al. 2015). We grouped features at superclass level via NPClassifier (Kim et al. 2021) in SIRIUS/CANOPUS (Dührkop et al. 2019, 2021) and assessed the difference in metabolite composition of nematode-treated roots using PERMANOVA via ‘adonis2’ function in R-package vegan, with Euclidean distance.

The nematode attraction assay was analyzed using *Friedman rank sum-test* via the Friedman.test function in rstatix package (Kassambara, 2024) followed by post-hoc pairwise *Wilcoxon test* with *Bonferroni correction*. Differences in nematode root penetration, as well as in GSLs, GSL breakdown products and gene expression data, were identified using generalized linear model (GLM) with the lme4 package in R (Bates et al. 2015). Data were Log-transformed to attain normal distribution when needed. In datasets that do not follow a Gaussian distribution based on visual (histogram) and statistical (*Shapiro-Wilk Test*) assessments, specific GLM distribution functions were used depending on the characteristics of each dataset (Bates et al. 2015). Estimated marginal means (emmeans) in R was used for post-hoc comparison. Figures and plots were generated in R with ggplot2 or in MetaboAnalyst 6.0 and were optimized for publication in Inkscape 1.1.1 (3bf5ae0d25, 2021-09-20, inkscape.org).

## Results

### Effects of Concurrent Nematode Infection on Root Metabolic Profiles

**Fig. 1:**
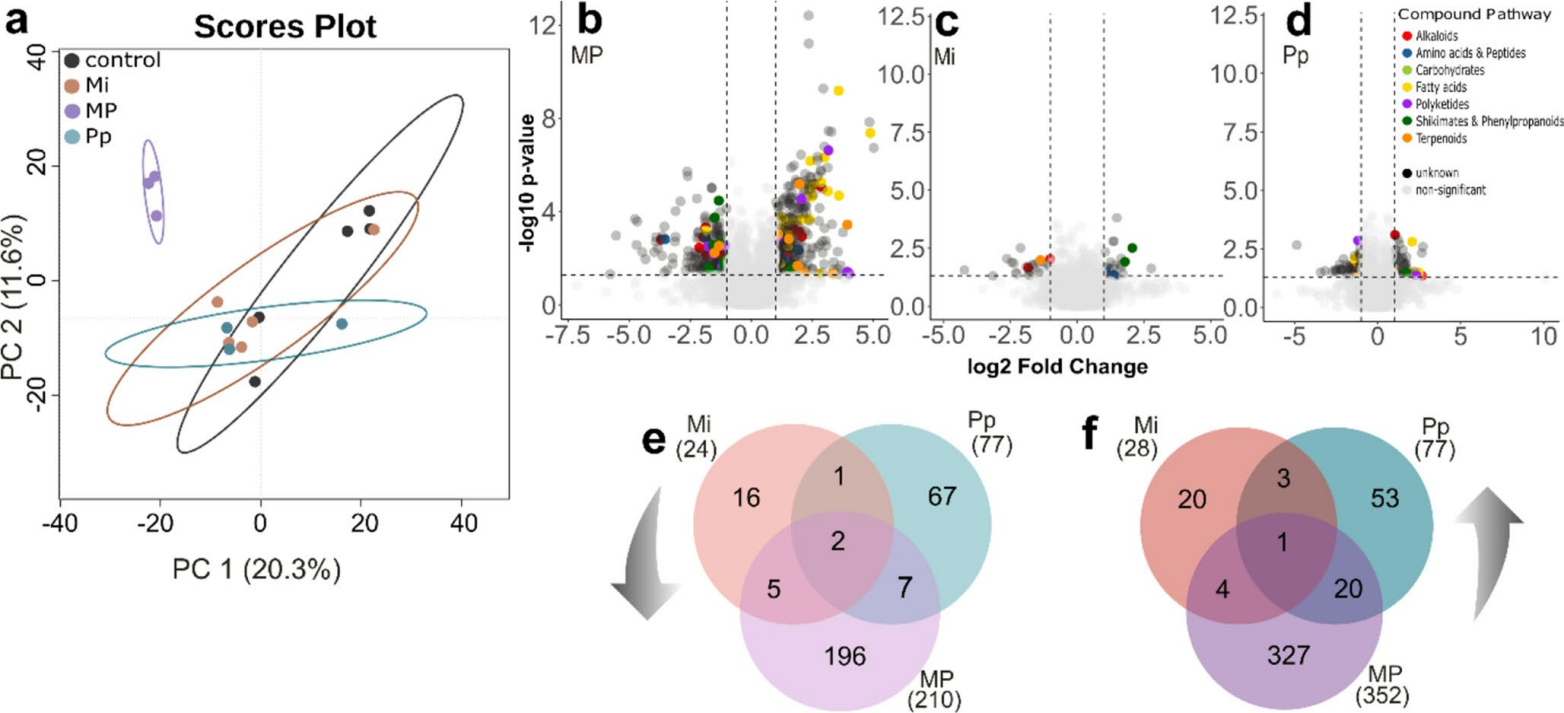
Metabolic profile of *Brassica nigra* roots (**a-f**), ten days after single (Mi, Pp) or dual (MP) nematode treatments. Metabolic profiles were constructed based on LC-qToF-MS/MS analyses in positive ionization mode. (**a**) Separation of root metabolic profiles of nematode-treated plants and mock-inoculated control plants shown in 2D scores plot following *Principal Component Analysis* (PCA). Points represent replicates per treatment group (n_control, Mi_ = 5; n_Pp, MP_ = 3), percentages at the axes indicate the variation explained by each component, ellipses indicate 95% confidence interval. Volcano plots (**b**,** c**,** d**) showing differentially abundant metabolites in the roots of (**b**) MP-, (**c**) Mi-, and (**d**) Pp-infested versus control plants. Metabolites were considered differentially abundant (DA) based on the following thresholds: fold change > 2.0, *P* ≤ 0.05 (t-test). The colored points represent metabolite groups classified via NPClassifier. The number of unique and shared DA metabolites are summarized in Venn Diagrams (**e-f**). These diagrams show the number of down- (**e**) and up- (**f**) regulated metabolites in the roots. The complete list of DA metabolites is given in Table S3-a-c. Abbreviations: Mi = *Meloidogyne incognita*, Pp = *Pratylenchus penetrans*, MP = *M. incognita* + *P. penetrans*

*Principal component analysis* (PCA) showed that the root metabolic profile of MP-infected plants differed distinctly from that of control, Mi- and Pp-infected plants (Fig. 1a, Table S2). This concurs with the observation that the roots of MP-treated plants had the most differentially accumulated (DA) features, both for up (352)- and down (210)-regulated features (562 in total; Fig. 1b, e-f). The roots of Mi-treated plants had the least up- (28) or down- (24) regulated features (Fig. 1c, e-f), followed by Pp-treatment (77 down- and 77 up-regulated features; Fig. 1d, e-f). A complete list of DA features is given in Table S3-a-c. Some DA features could be annotated using our in-house library. Among these annotated features, we found that Pp treatment uniquely up-regulated sinigrin levels (Fig. S2; Table S3-b). MP treatment uniquely down-regulated desulfo-4-hydroxyglucobrassicin and desulfo-glucobrassicin, and uniquely up-regulated cis-12-oxo-phytodienoic acid (OPDA) (Fig. S2; Table S3-c).

**Fig. 2:**
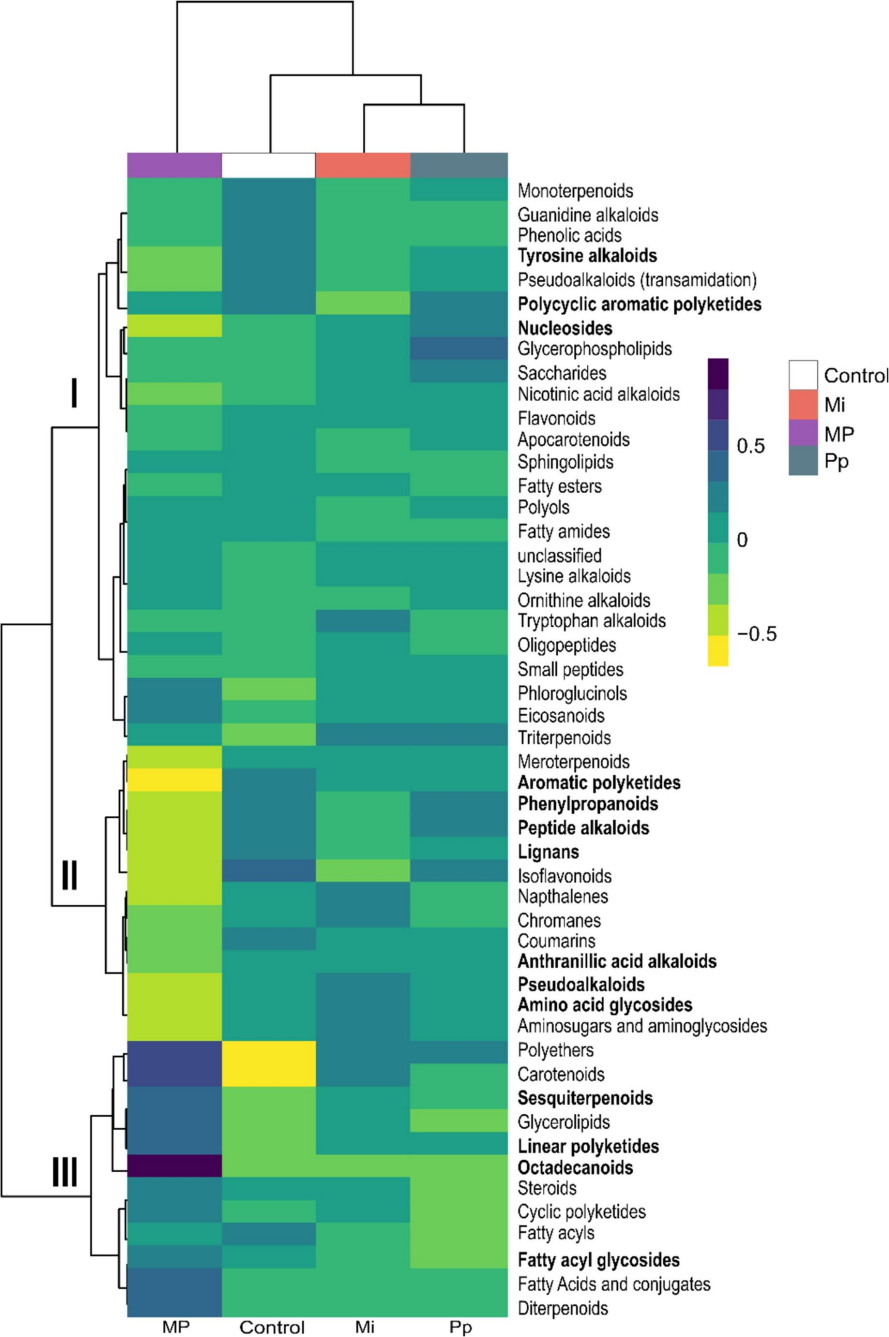
Heatmap showing clustering of features grouped according to NPClassifier superclass from untargeted secondary metabolite analysis of *Brassica nigra* roots from plants infected with single (Mi, Pp) and dual nematode (MP) species, as well as uninfected (control) plants. Clustering was based on *Euclidean distance* and *Ward algorithm* methods. Dark blue and yellow gradient represent the relative abundance of compounds based on log_10_-transformed and Pareto-scaled peak intensities, shown as average per treatment (*n* = 3–5). Dark blue indicates higher relative intensity; yellow indicates lower relative intensity. Compound groups written in bold letters are significantly different at *P* < 0.05. Abbreviations: Mi = *Meloidogyne incognita*, Pp = *Pratylenchus penetrans*, MP = *M. incognita* + *P. penetrans*

We used the features that could be assigned to superclass level (858 of 5919 features) to analyze differences in the root metabolic composition of control and nematode-treated plants. A PERMANOVA showed that nematode treatment significantly affected root chemical composition (*F* = 1.941, *df* = 3, *P* = 0.004; Fig. 2, Table S4). The nematode treatments explained approximately 32.7% of the variation in the dataset. The grouped features could be divided into three clusters (Fig. 2). The first cluster (labelled I in Fig. 2) included compound groups that are different in one or two treatment groups (Fig. 2). For example, tyrosine alkaloids (*F* = 5.268, *df* = 3, *P* = 0.023) were reduced in Mi- and MP-treated plants, but unchanged in Pp-infected plants. Nucleoside levels (*F* = 3.608, *df* = 3, *P* = 0.05) were slightly increased in Pp-infected plants and slightly reduced in MP-treated plants, whereas polycyclic aromatic polyketides (*F* = 4.731, *df* = 3, *P* = 0.028) were reduced in Mi-treated plants. The second cluster is characterized by reduced peak intensity in MP-treated plants (Fig. 2-II). Among these are defense-related compound classes in the shikimate pathway, such as lignans (*F* = 6.05, *df* = 3, *P* = 0.007) and phenylpropanoids (*F* = 5.517, *df* = 3, *P* = 0.01). Similarly, aromatic polyketides (*F* = 9.271, *df* = 3, *P* = 0.002), peptide alkaloids (*F* = 11.528, *df* = 3, *P* = 0.001), anthranilic acid alkaloids (*F* = 3.303, *df* = 3, *P* = 0.05), pseudoalkaloids (*F* = 5.886, *df* = 3, *P* = 0.01), and amino acid glycosides (*F* = 4.895, *df* = 3, *P* = 0.031) were also significantly reduced in response to MP treatment. The NPClassifer-based classification assigned many GSLs to the amino acid glycoside class (Table S3-d). The third cluster is composed of compound groups with increased peak intensities in response to MP treatment. This includes sesquiterpenoids (*F* = 5.446, *df* = 3, *P* = 0.021), linear polyketides (*F* = 4.653, *df* = 3, *P* = 0.024), fatty acyl glycosides (*F* = 8.650, *df* = 3, *P* = 0.003), and octadecanoids (*F* = 66.028, *df* = 3, *P* = 0.004). Specifically, MP treatment uniquely accumulated eight features classified as octadecanoids (Fig. S2) and uniquely reduced four features classified as lignans (Fig. S2). A complete list of PERMANOVA results can be found in Table S4.

In addition, we analyzed the expression levels of common marker genes related to defense signaling pathways, such as *PR1*, *PAL1*, and *ERF1*. The jasmonate/ethylene regulator *ERF1* was significantly down-regulated in all of the nematode-infected roots compared to the roots of control plants (GLM_Gamma_: χ^2^ = 23.114, *df* = 3, *P* < 0.001; Fig. S3). With marginal statistical significance, the SA-responsive gene *PR1* was down-regulated in MP-treated roots compared to untreated roots (GLM_Gamma_: χ^2^ = 6.024, *df* = 3, *P* = 0.111, Fig. S3). *PAL1*, however, was not differentially expressed by any of the nematode treatments (LM_Gaussian_: *F* = 0.76, *df* = 3, *P* = 0.540). Unfortunately, our analyses of the JA-related gene yielded low expression values and therefore could not be reliably interpreted.

Altogether, these results suggest that metabolic changes in response to concurrent infection by *M. incognita* and *P. penetrans* differed from the changes in response to each PPN species alone. The differences are reflected in the levels of many defense-related compound classes such as lignans, phenylpropanoids, octadecanoids, and GSLs.

### Nematode-induced Changes in Different Classes of Root GSL

Based on the metabolic analyses, where we found the class amino acid glycosides, comprising many GSLs, significantly regulated, we performed targeted analyses of GSLs and their breakdown products. In addition, we analyzed the expression of representative GSL biosynthesis genes. The composition and relative abundance of GSL and breakdown products detected in the roots via targeted analysis is given in Table S5.

**Fig. 3:**
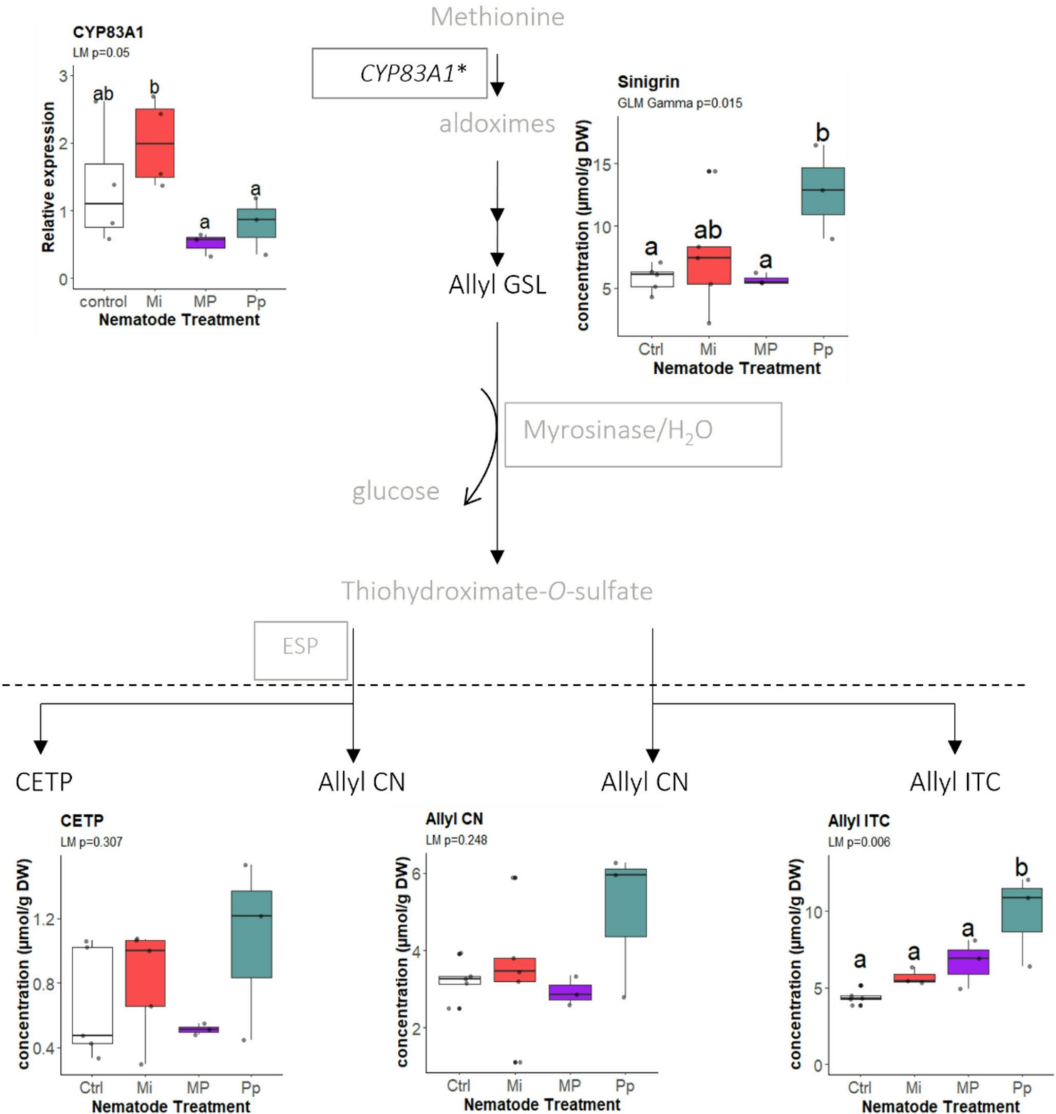
Contents of the aliphatic glucosinolate sinigrin (allyl glucosinolate) and its breakdown products in *Brassica nigra* roots, including the relative expression of CYP83A1 gene as they occur in the aliphatic glucosinolate metabolism pathway. Boxes and names in gray are part of the pathways that were not measured in the experiment. Compounds below the broken line are breakdown products. The biological replicates per treatment (n_control, Mi_ = 5; n_Pp, MP_ = 3) are represented by points in the boxplots. Boxplots labeled with different letters are significantly different as per the estimated marginal means (EMMEANS) at *P* < 0.05. Abbreviations: Mi = *Meloidogyne incognita*, Pp = *Pratylenchus penetrans*, MP = *M. incognita* + *P. penetrans*, CETP = 1-cyano-2,3-epithiopropane, CN = cyanide, ITC = isothiocyanate. *CYP83A1 also occurs in the benzenic glucosinolate biosynthesis pathway

In both targeted and untargeted analyses, we found that the levels of sinigrin were significantly higher in Pp-infected plants compared to control and MP-treated plants (GLM_Gamma_: χ^2^ = 10.511, *df* = 3, *P* = 0.015, Fig. 3). In line with this observation, we found a significant increase of the degradation product, allyl ITC, in roots of Pp-treated plants compared to other treatments (LM_Gaussian_: *F* = 7.563, *df* = 3, *P* = 0.006; Fig. 3). The other breakdown products, 1-cyano-2,3-epithiopropane (CETP) and allyl CN showed a similar pattern, though the differences were not statistically significant. Interestingly, the expression of *CYP83A1*, involved in the synthesis of sinigrin, was significantly upregulated by Mi infection (LM_Gaussian_: *F* = 3.67, *df* = 3, *P* = 0.05; Fig. 3) compared to Pp- and MP-infections.

**Fig. 4:**
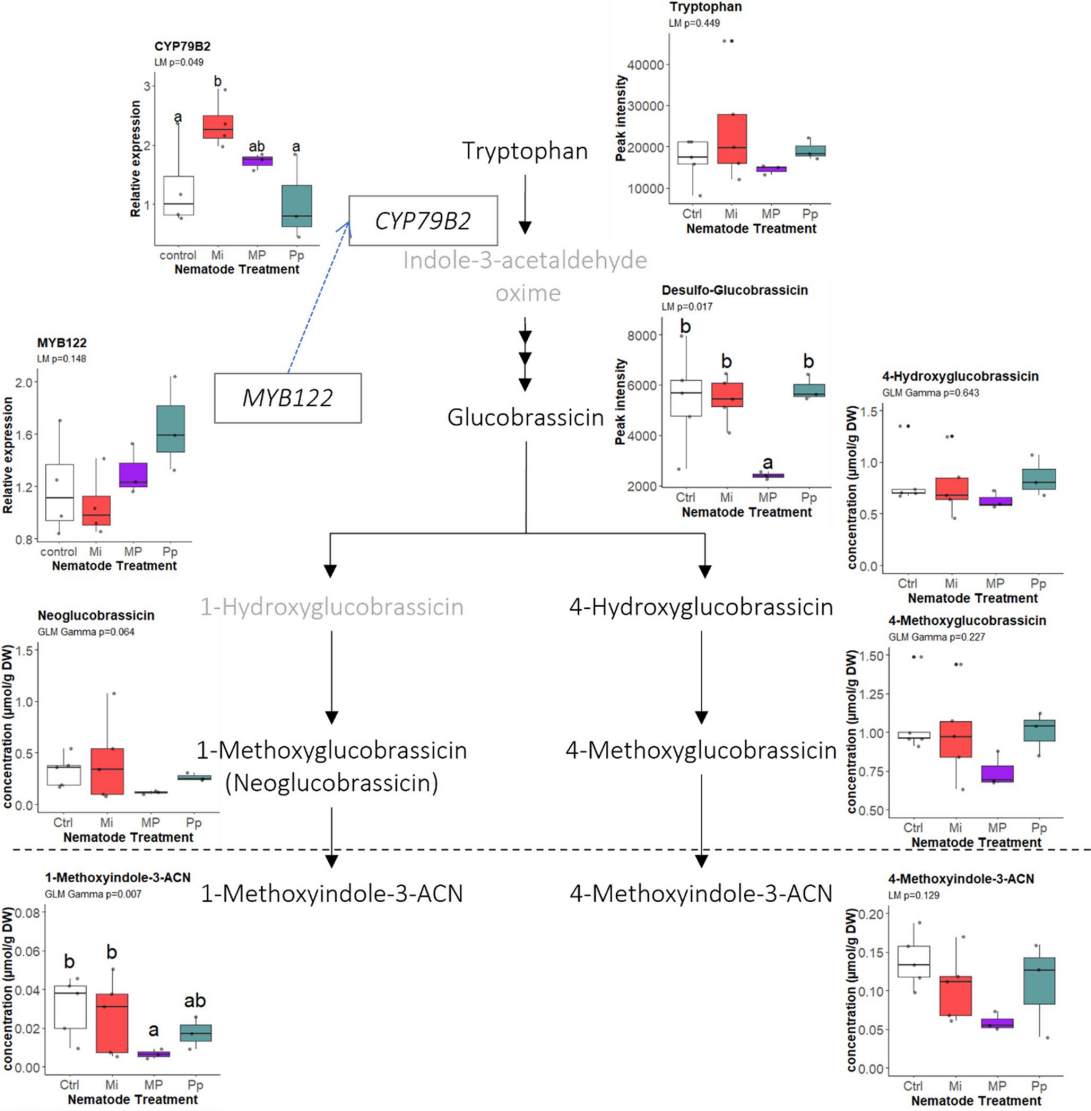
Contents of tryptophan, indole glucosinolates, and their breakdown products in *Brassica nigra* roots, including the relative expressions of CYP79B2 gene and the transcription factor MYB122 as they occur in the indole glucosinolate metabolism pathway. Boxes and names in gray are parts of the pathway that were not measured in the experiment. Metabolites below or onto the right of the broken line are breakdown products. The biological replicates per treatment (n_control, Mi_ = 5; n_Pp, MP_ = 3) are represented by points in the boxplots. Boxplots labeled with different letters are significantly different as per the estimated marginal means (EMMEANS) at *P* < 0.05. Abbreviations: Mi = *Meloidogyne incognita*, Pp = *Pratylenchus penetrans*, MP = *M. incognita* + *P. penetrans*

For the indole GSLs, particularly for glucobrassicin (LM_Gaussian_: *F* = 5.098, *df* = 3, *P* = 0.017) and the neoglucobrassicin degradation product, 1-methoxyindole-3-acetonitrile (GLM_Gamma_: χ^2^ = 12.129, *df* = 3, *P* = 0.007), we found that they are significantly reduced in the roots of MP-treated plants compared to other treatment groups (Fig. 4). Showing a similar pattern, although not statistically significantly so, were the precursor amino acid, tryptophan (LM_Gaussian_: *F* = 0.947, *df* = 3, *P* = 0.449), the GSLs downstream in the process, neoglucobrassicin (LM_Gamma_: χ^2^ = 7.262, *df* = 3, *P* = 0.064), 4-hydroxyglucobrassicin (GLM_Gamma_: χ^2^ = 1.674, *df* = 3, *P* = 0.643), and 4-methoxyglucobrassicin (GLM_Gamma_: χ^2^ = 4.341, *df* = 3, *P* = 0.227), as well as the other indole GSL breakdown product, 4-methoxyindole-3-ACN (LM_Gaussian_: *F* = 2.305, *df* = 3, *P* = 0.129). Moreover, *CYP79B2* was significantly upregulated by Mi-treatment (LM_Gaussian_: *F* = 3.74, *df* = 3, *P* = 0.049) compared to control and Pp-infected plants, while the transcription factor *MYB122* was not differentially expressed by any of the nematode treatments (LM_Gaussian_: *F* = 2.27, *df* = 3, *P* = 0.143) (Fig. 4).

**Fig. 5:**
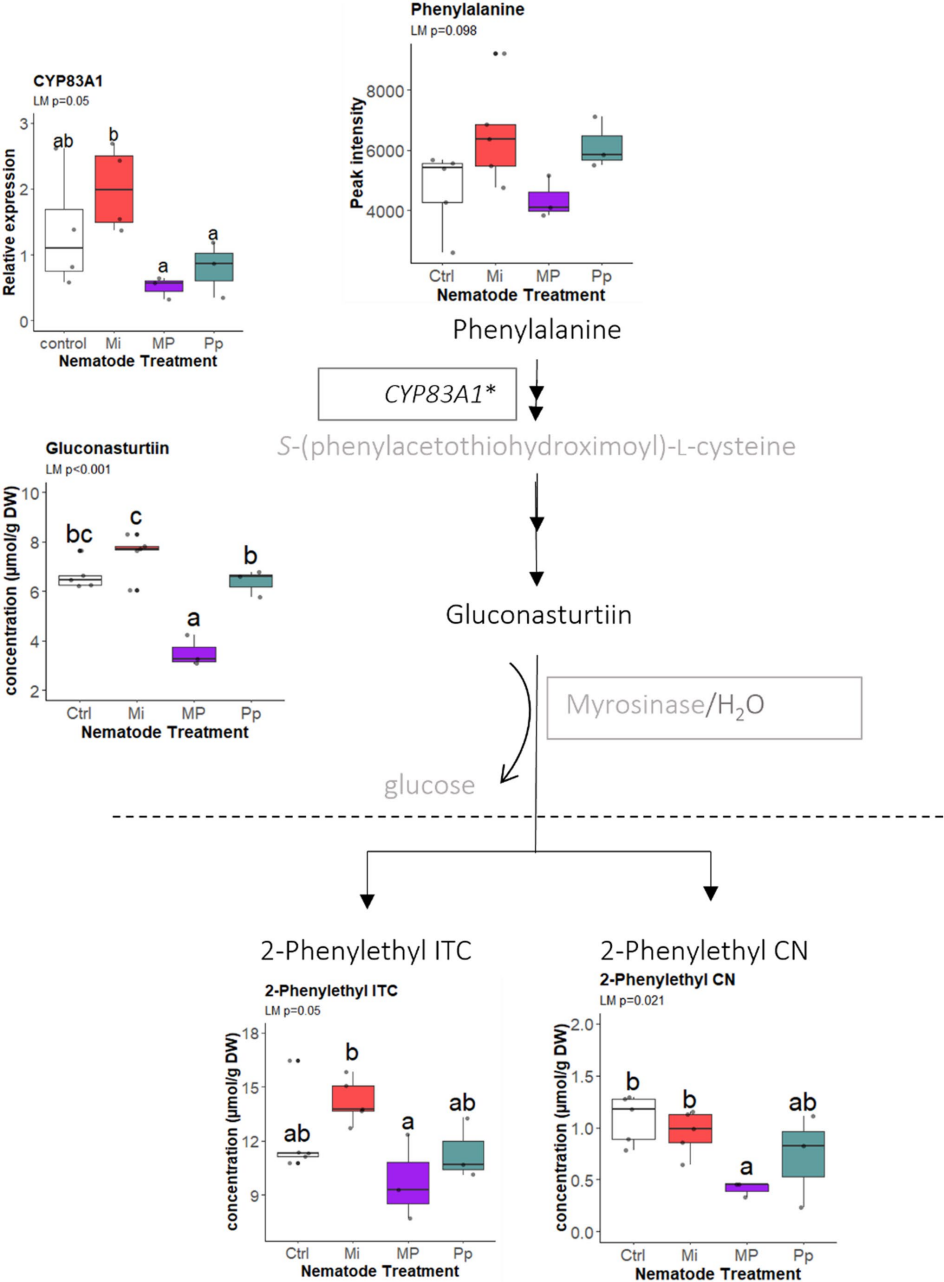
Contents of phenylalanine, the glucosinolate gluconasturtiin, and the corresponding breakdown products in *Brassica nigra* roots, including the relative expressions of CYP83A1 gene as they occur in the benzenic glucosinolate metabolism pathway. Boxes and names in gray are parts of the pathway that were not measured in the experiment. Compounds below the broken line are breakdown products. Boxplots labeled with different letters are significantly different as per the estimated marginal means (EMMEANS) at *P* < 0.05. The biological replicates per treatment (n_control, Mi_ = 5; n_Pp, MP_ = 3) are represented by points in the boxplots. Abbreviations: Mi = *Meloidogyne incognita*, Pp = *Pratylenchus penetrans*, MP = *M. incognita* + *P. penetrans*, CN = cyanide, ITC = isothiocyanate. *CYP83A1 also occurs in the aliphatic glucosinolate biosynthesis pathway

Similar to the indole GSLs, the levels of the benzenic GSL gluconasturtiin (LM_Gaussian_: *F* = 22.128, *df* = 3, *P* < 0.001) and its degradation products, 2-phenylethyl ITC (LM_Gaussian_: *F* = 3.477, *df* = 3, *P* = 0.05) and 2-phenylethyl CN (LM: *F* = 4.753, *df* = 3, *P* = 0.021), had significantly lower levels in MP-treated plants (Fig. 5). Gluconasturtiin levels were significantly higher in Mi- (*P* < 0.001) and Pp-treated plants (*P* < 0.001) compared to MP-treated plants. In addition to its role in aliphatic GSL biosynthesis, the *CYP83A1* gene is also involved in benzenic GSL biosynthesis. *CYP83A1* was significantly downregulated in MP- and Pp-treated plants compared to Mi-treated plants (LM_Gaussian_: *F* = 3.67, *df* = 3, *P* = 0.05) (Fig. 5). The expression pattern of *CYP83A1* was more similar to changes in this pathway than those in the aliphatic glucosinolate (sinigrin) pathways (Fig. 3).

These results showed that single-species and concurrent nematode infections led to distinct changes in GSL classes. In MP-treated plants, the levels of indole GSL and their breakdown products were reduced compared to Mi and Pp plants. Both MP and Mi treatments altered the benzenic GSL, with marginally significant increased levels in response to Mi, yet significantly reduced levels in response to MP. Infection with Pp resulted in increased levels of sinigrin and its conversion products.

### Simultaneous Infection Affected the Early Performance of Each Nematode Species

**Fig. 6:**
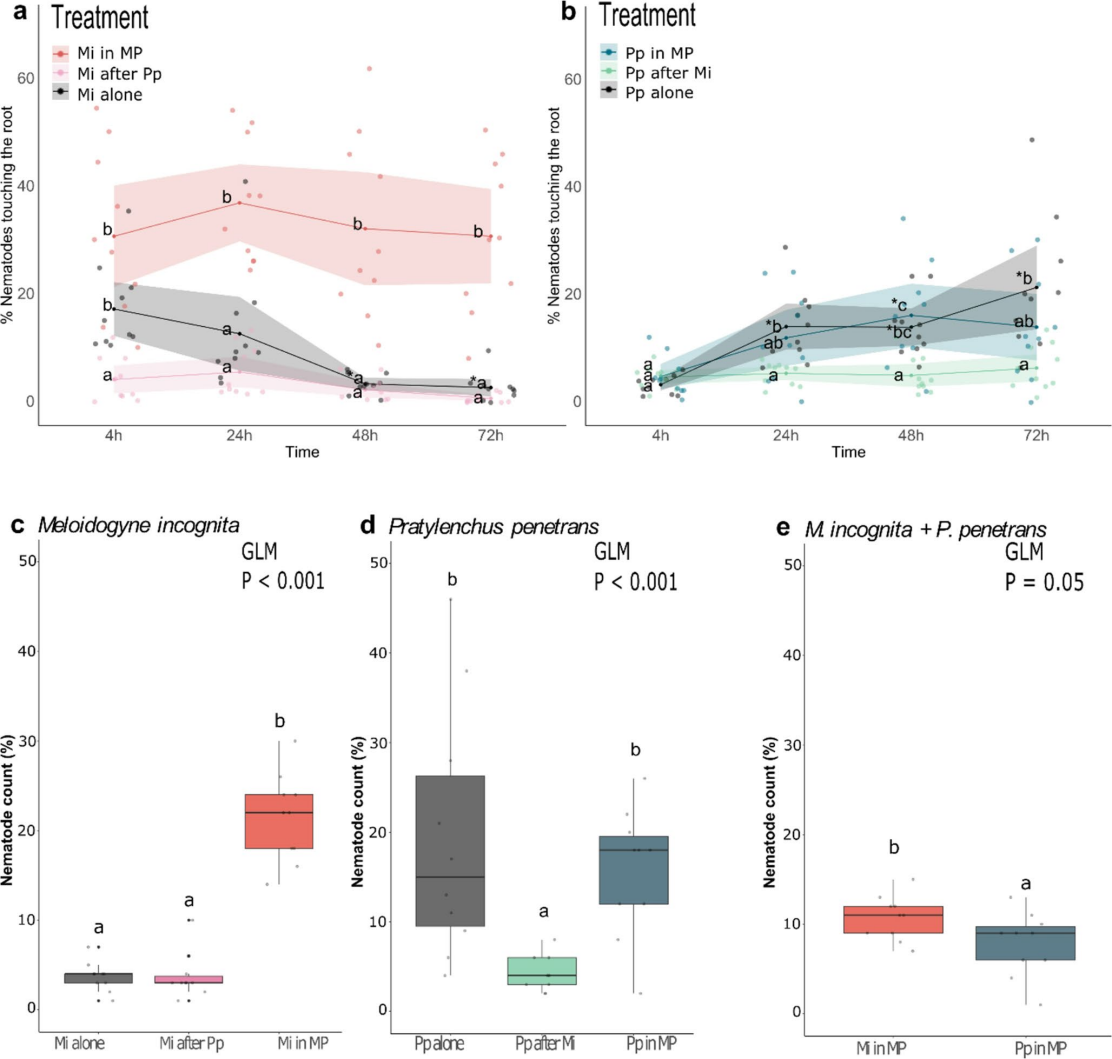
In vitro nematode performance assay showing nematode attraction (**a-b**) and early penetration in a Pluronic gel plate (**c-e**). The PPNs of interest were inoculated alone (Mi, Pp); inoculated two days after the other (Pp after Mi, Mi after Pp) or inoculated together (Mi in MP, Pp in MP). a and b show the proportion of nematodes Mi or Pp found touching the root at 4, 24, 48 and 72 h after inoculation in Pluronic gel plates. The connected dark points denote the mean per treatment group per time point. Means that are marked with * are statistically different from the first counting time (4 h) within each treatment group. Means connected by different letters denote significant difference of treatments per designated time point based on *Wilcoxon’s pairwise test* at *P* < 0.05 following *Friedman Rank Sum test*. The colored points represent the biological replicates per treatment group, *n* = 10. The spread of ribbons denotes 95% confidence interval. c-e shows the number of nematodes that were found inside *B. nigra* roots at three-day post inoculation via acid fuchsin staining. c-d presents a comparison of Mi (c) and Pp (d) penetration under different conditions: when inoculated concurrently, alone, or sequentially after the other species. For single-species plates (Mi alone, Mi after Pp, Pp alone, and Pp after Mi) 100 individuals were added to each plate. For dual-species plates (Mi in MP and Pp in MP) 50 individuals of each species were added (total of 100 nematodes per plate). The nematode counts were normalized to percentages by dividing the actual count of species X by the numbers of species X added to the plate (either 100 or 50). e illustrates the percentages of Mi and Pp that penetrated the roots of plants with concurrent PPN infection. The datasets were analyzed with the *Generalized Linear Model* (GLM). Boxplots labeled with different letters are significantly different as per the estimated marginal means (emmeans) at *P* < 0.05. Boxplot legend: The box represents the interquartile range with the upper line representing the 75th percentile and the lower line representing the 25th percentile. The middle line represents the median. The whiskers extend to the (upper) maximum and (lower) minimum values, illustrating the overall spread of the data within one treatment. Black dots beyond the whiskers represent the outliers. Points represent the number of biological replicates per treatment, *n* = 10. Abbreviations: Mi = *Meloidogyne incognita*, Pp = *Pratylenchus penetrans*, MP = *M. incognita* + *P. penetrans*

The attraction of *M. incognita* to uninfected roots differed from that of roots that were pre-infested or simultaneously infested with *P. penetrans* (Fig. 6a, Friedman χ^2^ = 75.685, *df* = 4, *P* < 0.001; Fig. 6a). Over time, fewer *M. incognita* touched the roots when the plants were pre-inoculated with Pp, in particular, compared to MP (M in MP, Wilcoxon test: 4 h *P* = 0.018, 24 h *P* = 0.018, 48 h *P* = 0.006, 72 h *P* = 0.017; Fig. 6a). Similarly, fewer *P. penetrans* touched *B. nigra* roots when plants were pre-infested with Mi (Fig. 6b, Friedman χ^2^ = 85.573, *df* = 4, *P* < 0.001), especially when compared to plants where *P. penetrans* was inoculated alone. By the end of the counting period, more *M. incognita* were touching the roots of MP-inoculated plants than in plants where they were the only nematode (Mi), or when they were inoculated after *P. penetrans* (Mi after Pp; Fig. 6a). In contrast, significantly more *P. penetrans* were touching the roots when they were the only nematode in the plate (Pp) than when inoculated after *M. incognita* (Pp after Mi; Fig. 6b).

Inoculating Mi after or with Pp affected the penetration success of Mi at 3 dpi (GLM_Gamma_: χ^2^ = 94.512, *df* = 2, *P* < 0.001; Fig. 6c). We found more *M. incognita* in roots of the MP-treated plants compared to Mi plants (EMMEANS: *P* < 0.001) or to roots pre-infected with *P. penetrans* (Mi after Pp) (EMMEANS: *P* < 0.001; Fig. 6c). Single and concurrent inoculations also differentially affected the *P. penetrans* penetration (GLM_Gamma_: χ^2^ = 31.21, *df* = 2, *P* < 0.001; Fig. 6d). The numbers of *P. penetrans* found in the roots of dual-infected plants (P in MP) were significantly higher than in plants with prior *M. incognita* infection (Pp after Mi, EMMEANS: *P* = 0.001; Fig. 6d). There were also significantly more *P. penetrans* found in the roots of singly-infected plants (Pp alone) compared to Mi pre-infested plants (EMMEANS: *P* < 0.001; Fig. 6d). When both nematodes were inoculated at the same time (MP), we found significantly more *M. incognita* than *P. penetrans* in the roots (LM_Gaussian_: *F* = 4.5134, *df* = 1, *P* = 0.0477; Fig. 6e).

Overall, these results showed that concurrent infection distinctly affects the early infection success of each nematode species, whereby the effects depended on the species and sequence of infection.

## Discussion

To understand how concurrent nematode infection affected the performance of each nematode species, we examined how infection by *M. incognita* and *P. penetrans*, alone and together, affected root metabolic profiles, particularly GSL and their breakdown products. Overall, concurrent infection led to a distinctly different root metabolic profile compared to that of single-species infections. This was mainly due to differences in defense-related compounds, such as lignans, phenylpropanoids, and GSLs. The different GSL classes responded individually to the different nematode treatments; Mi treatment increased benzenic GSLs, Pp treatment increased aliphatic GSLs, and MP treatment reduced indole GSLs. Furthermore, we found that *M. incognita* performs better when co-inoculated with *P. penetrans*, whereas *P. penetrans* performed worse when having to colonize a *M. incognita* infected plant.

### Concurrent PPN Infection Affected Root Metabolome Differently than Single-species Infection

In natural environments, plants are often simultaneously attacked by multiple herbivores, resulting in complex interactions between signaling pathways (Bonnet et al. 2017; van Dam et al. 2018; Mbaluto et al. 2021). When herbivores with contrasting feeding strategies attack, the response to one herbivore can antagonize the response to another due to hormonal crosstalk or defense compound interaction (Pieterse et al. 2009; Bonnet et al. 2017; Mbaluto et al. 2021). For example, simultaneous attacks by aphids and caterpillars can weaken JA-mediated defenses, making plants more vulnerable to caterpillar damage (Soler et al. 2012; Ali and Agrawal 2014). Our study examined the root metabolic changes in response to concurrent infection of two nematodes with contrasting feeding strategies – the sedentary endoparasite, *Meloidogyne incognita* and the migratory endoparasite, *Pratylenchus penetrans.* First, we hypothesized that each nematode species would elicit a specific root metabolic response. Our results showed that the overall root metabolic profiles of *M. incognita* and *P. penetrans* infected plants did not significantly differ from each other, nor did they differ from that of uninfected control plants (Fig. 1a, Table S2). However, when looking into the differentially accumulated (DA) features in nematode-infected vs. control roots, we found several DA features responding to *M. incognita* and *P. penetrans*, with more DA compounds uniquely responding to each species, than DAs shared among these treatment groups (Fig. 1e-f). *Pratylenchus penetrans* uniquely up-regulated sinigrin levels, which is a typical response to tissue damage in *Brassica* spp. (Traw 2002). The continuous tissue damage caused by the migratory *P. penetrans* can cause increased level of the toxic allyl ITC. This response is less expected of *M. incognita* infections, as early *Meloidogyne* spp. infection typically causes less tissue damage (Gheysen and Mitchum 2011; van Dam et al. 2018). Moreover, in the infection process, *Meloidogyne* spp. secrete effectors to down-regulate plant defense responses (Gheysen and Mitchum 2011; Kyndt et al. 2012a).

We further hypothesized that concurrent infections of *M. incognita* and *P. penetrans* attenuate changes in the root metabolic profile. Concurrent infection resulted in a distinct root metabolic profile (Fig. 1a). Other than expected, double infected plants showed the highest numbers of significantly upregulated and downregulated DA features (Fig. 1b), indicating a more complex and intense metabolic response. Interestingly, the distinct changes in root metabolic profiles could be predominantly attributed to the differential accumulation of several defense compounds in response to MP treatment (Fig. 2). This may suggest that dual infection may have amplified the plant’s defense responses, resulting in the simultaneous regulation of multiple defense-related pathways. The distinguished increase of OPDA levels and those of other octadecanoids during dual infection suggests that the octadecanoid pathway may have been involved in enhancing the production of defensive metabolites (León and Sánchez-Serrano 1999; Papazian et al. 2019). On the other hand, dual infection also reduced the levels of lignans and phenylpropanoids, two distinct groups of compounds with critical roles in plant defense (Dixon et al. 2002; Ražná et al. 2021). Both compound classes have been implicated in plant responses to the RKN *M. javanica* in tomatoes, as demonstrated by the upregulation of transcripts encoding for enzymes involved in their biosynthesis (Kamali et al. 2022). Compounds classified as alkaloids, i.e., peptide alkaloids, aromatic alkaloids, anthranilic acid alkaloids, as well as aromatic polyketides were also reduced in MP-treated roots. The reduction of these compounds suggests that plant defense response to single nematode species may be compromised under double infection. It should be noted that, because root damage was not yet visible at the sampling timepoint, we could not compare damage levels between treatments. Potential differences in damage levels due to direct or indirect interactions between the two nematode species in the early stages may also influence plant metabolic responses in double infected plants.

Moreover, amino acid glycosides, a class in which several GSLs are found, were also significantly reduced in response to MP treatment. GSLs are key defense compounds in *Brassica* plants and are known for their defensive role against herbivores, including nematodes (Potter et al. 2000). This motivated us to perform targeted analysis of GSLs and GSL breakdown products to further understand their role in the response to dual and single-species nematode infections.

#### Nematode Infection Affects Specific GSL Metabolic Pathways

Our results demonstrated that single-species and concurrent infections by *M. incognita* and *P. penetrans* led to distinct alterations in the root GSL profile of *B. nigra*. The ‘mustard oil bomb’ mechanism, where tissue damage triggers the GSL hydrolysis by the enzyme myrosinase (Abdel-Massih et al. 2023), produces toxic breakdown products that can affect insects, microbes, and nematodes (van Dam et al. 2009; Eugui et al. 2022; Sontowski et al. 2022; Chekanai et al. 2024).

Allyl GSLs were induced by *P. penetrans* (Fig. 2). This suggests an induced response that may be associated with the direct and extensive mechanical damage inflicted by *P. penetrans* on root tissues. Allyl ITC, in particular, was shown to reduce the motility and increase the mortality of *P. penetrans* in a time- and dose-dependent manner (Chekanai et al. 2024). In MP-infected plants, however, sinigrin and allyl ITC levels were significantly reduced compared to that of Pp-treated plants. This reduction suggests that the presence of *M. incognita* may have interfered with the sinigrin-mediated response of *B. nigra* towards *P. penetrans.* Follow-up bioassays using mutants impaired in the production of sinigrin or allyl-ITC in their roots, could further elucidate the role of sinigrin and its breakdown products in the defense against *P. penetrans.*

Benzenic GSLs were also reduced in MP-treated plants, whereas these compounds were slightly higher in Mi-treated plants (Fig. 5). Indeed, gluconasturtiin and 2-phenylethyl ITC are among the GSLs deemed effective in suppressing nematode populations (Potter et al. 2000; Eugui et al. 2022), in particular those of *Pratylenchus* spp. (Potter et al. 1998, 2000). Our results thus align with previous reports that gluconasturtiin and 2-phenylethyl ITC play a role in mediating plant responses to nematode infections (Potter et al. 2000; Eugui et al. 2022).

Concurrent infection reduced the less abundant indole GSLs, particularly glucobrassicin and 1-methoxyindole-ACN (Fig. 4). Root-knot nematode feeding leads to significant changes in plant root tissue, starting with the establishment of feeding sites that result in the formation of giant cells, creating root galls (Gheysen and Mitchum 2011; Mbaluto et al. 2021). Transcriptomic analysis of giant cells obtained via laser dissection has shown that the indole GSL biosynthesis gene, *CYP79B2* was down-regulated in the giant cells but up-regulated in surrounding vascular tissues (Barcala et al. 2010). This shows that the metabolic route involving indole GSL can be among the defense pathways responding to RKN infection and may be manipulated by the nematode for successful feeding. Our results point to a similar direction. We also found a significant upregulation of *CYP79B2* in *M. incognita-*infected roots (Fig. 4). Indole GSLs, in particular, are known for their anti-pathogen effects (Clay et al. 2009). Increased indole GSL levels have also been correlated with response to aphid feeding (Kim and Jander 2007; Pajar et al. 2024). Aphids and nematodes, particularly, RKN, share similarities regarding their interactions with host plants. Both organisms use effectors to manipulate plant metabolic processes while creating their respective feeding sinks (Gheysen and Mitchum 2011; Züst and Agrawal 2016). Given this similarity, it is not surprising that indole GSL could be one of the metabolites that respond to *M. incognita* infection. If indole GSLs indeed confer resistance to RKN, then the reduction of glucobrassicin and 1-methoxyindole-3-ACN in MP-treated plants may have potentially benefited *M. incognita*, which correlates to better early infection performance compared to single-species inoculation. However, further evaluation of the specific effects of indole GSLs and their breakdown products is needed to assess their defensive role against nematodes.

Our results also showed that even the least abundant GSL breakdown products varied between treatments. In particular, we found a significant reduction of the less abundant 1-methoxyindole-3-ACN (Fig. 4) and 2-phenylethyl CN (Fig. 5) in the roots with MP treatment compared to Mi-treated and control plants. Due to their high GSL concentrations, several *Brassica* species were successfully used in biofumigation (Dahlin and Hallmann 2020; Eugui et al. 2022). Biofumigation incorporates chopped plant materials into the soil, releasing toxic GSL breakdown products with nematocidal effects. This process significantly reduced nematode populations (Potter et al. 1998; Eugui et al. 2022), including over 60% reduction in galls and egg masses of *Meloidogyne* spp. in greenhouse settings (Oliveira et al. 2011; Curto et al. 2016).

While studies on the effect of GSL breakdown products mostly focused on the more abundant and biologically active ITCs, these results indicate that also nitriles/CNs may play a role in plant-PPN interactions.

#### Insights on the Relationship of *M. incognita* and *P. penetrans* in Concurrent Infection

We also considered the presence of nematodes in close proximity to the roots (touching the root, attraction) and inside the roots (penetration) as measures of early infection success. Our results suggest that *M. incognita* is more successful when co-inoculated with *P. penetrans*, while *P. penetrans* is unaffected by co-inoculation but was more successful in the absence of prior *M. incognita* infection (Fig. 6). This implies that concurrent PPN infection affects each nematode species differently. *Meloidogyne* spp. and *Pratylenchus* spp. were reported to behave antagonistically in several plant pathosystems (Gay and Bird 1973; Chapman and Turner 1975; Fontana et al. 2015), but there are not many studies showing the possible mechanisms for this antagonism, particularly in relation to plant-mediated responses. We found that MP treatment, among other changes in root secondary metabolite, reduced indole (Fig. 4) and benzenic (Fig. 5) GSLs, as well as their breakdown products. The reduction of specific GSL and their breakdown products, as well as the down-regulation of metabolites belonging to other defense-related classes, such as phenylpropanoids and lignans (Fig. S2, Table S4), suggest that the plant defenses are compromised under dual infection. The enhanced levels of OPDA and other octadecanoids (Fig. S2) in MP-treated plants suggest increased signaling in the JA pathway, which in turn may have suppressed the SA signaling pathway. Since *M. incognita* is more susceptible to SA-based defenses (Bonnet et al. 2017; Gheysen and Mitchum 2019), the metabolic changes in response to concurrent infection may have favored *M. incognita.* This is associated with the greater early infection success of *M. incognita* in concurrent infections compared to single-species infection (Fig. 6). This is, however, not the case for *P. penetrans*, whose early infection success did not significantly differ when it had been inoculated alone or with *M. incognita.*

We observed an overall decline in the number of nematodes in contact with the roots over time, except in the Pp and Pp in MP treatment groups. The decline is likely due to the fact that *M. incognita* J2 rapidly penetrate the roots and thus are not visible outside anymore. To confirm this, we stained the roots after counting at 72 h. The stained roots of MP-treated plants showed that more *M. incognita* were able to enter the roots at 3 dpi compared to *P. penetrans* (Fig. 5). According to Mateille et al. (2020), PPN species may affect each other via competition when both species use the same limited resources. It is thus possible that, whereas the attenuated defense response favored *M. incognita* in MP treatments, *M. incognita* may have also outcompeted *P. penetrans* in exploiting the resources, such as nutrients and infection sites. Though we did not include root metabolic measurements of the Pp-after-Mi treatment, the results of the nematode performance assay showed that *P. penetrans* is negatively affected by prior *M. incognita* infection. Nevertheless, more bioassays, particularly using mutants impaired in previously mentioned signaling pathways, are needed to elucidate the underlying mechanism of nematode-nematode interactions in concurrent infection.

Taken together, we showed that the root metabolic profile of dual nematode-infected *B. nigra* is markedly different from those of plants infected by either *M. incognita* or *P. penetrans* alone. This suggests that the concurrent infection by both nematodes triggers a unique metabolic response that is not merely a combination of the individual responses to each nematode. This novel information generated from untargeted metabolic analysis could serve as basis for generating further hypotheses in exploring specific plant responses to single and dual nematode infections. In particular, different GSL classes responded differently to the nematode treatments. Considering the specificity of the GSL response, we suggest to further explore the specificity of GSL and GSL breakdown products as defenses against different plant parasitic nematode species. This knowledge has the potential to improve breeding programs aimed at enhancing nematode resistance, as well as enhance biofumigation strategies.

## Supplementary Information

Below is the link to the electronic supplementary material.

Supplementary file1 (XLSX 2.95 MB)

Supplementary file2 (PNG 105 KB)

## Data Availability

Data supporting this study are included in the manuscript and supplementary files.
