# Supplementary figures and images for: Dual and Single-species Nematode Infections Distinctly Modulate Defense Metabolism in Brassica nigra Roots
Source: J Chem Ecol. 2025 Sep 10;51(5):90. doi: 10.1007/s10886-025-01637-8 (PMC12423230; doi:10.1007/s10886-025-01637-8)

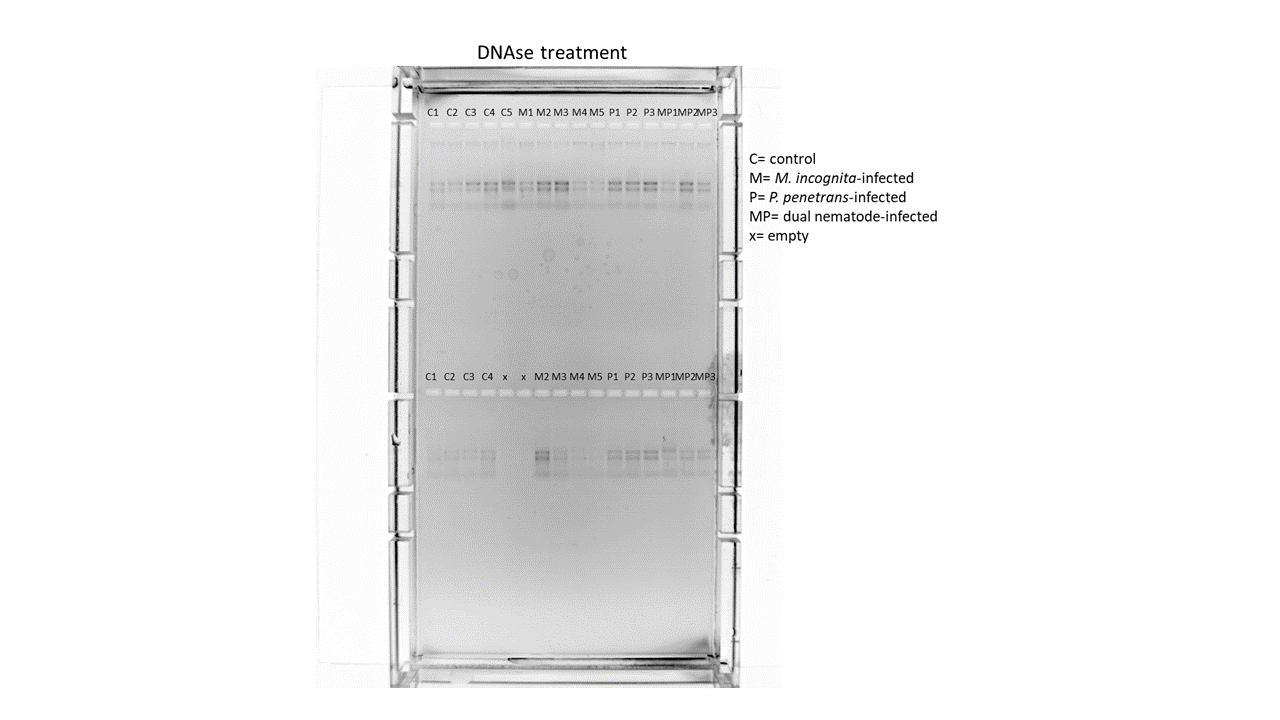

Supplement: Supplementary file 2 — Supplementary file2 (PNG 105 KB) [file 10886_2025_1637_MOESM2_ESM.png]
